# *P. micra* and *F. necrophorum*: Hepatic Abscesses in a Healthy Soldier

**DOI:** 10.1155/2022/5500365

**Published:** 2022-03-26

**Authors:** Samuel Strobel, Daniel Whitaker, Edwin Choi, Janelle Lindow, Kathryn Lago

**Affiliations:** ^1^Family Medicine Service, Womack Army Medical Center, 2817 Reilly Road, Fort Bragg 28310, NC, USA; ^2^Infectious Disease Service, Womack Army Medical Center, 2817 Reilly Road, Fort Bragg 28310, NC, USA

## Abstract

*Parvimonas micra (P. micra)* and *Fusobacterium necrophorum (F. necrophorum)* are two pathogens known to cause odontogenic and oropharyngeal infections. It is exceedingly rare for these bacteria to cause coinfection and even systemic infection. There is limited literature on liver abscesses and bacteremia involving *P. micra*. Most cases are found in elderly patients with associated gastrointestinal malignancy (24%) or laryngeal pharynx malignancy (28%). However, a substantial portion of described cases were unable to identify a source (36%). A 36-year-old, otherwise healthy male presented for fevers and chills for 2 weeks. After testing negative for initial infectious workup, including COVID-19 multiple times, he was found to have multiple liver abscesses which grew *P. micra* and *F. necrophorum*. This case highlights a rare coinfection of hepatic abscesses in an otherwise healthy young immunocompetent adult with a solitary dental caries, resulting in septic shock.

## 1. Introduction

*P. micra* is an anaerobic Gram-positive cocci [1]. *P. micra* is part of the normal flora of the oral cavity and gastrointestinal (GI) tract and is most often implicated in oral cavity infections [1]. *P. micra* bacteremia has been described originating from a variety of sources to include spondylodiscitis (29.6%), oropharyngeal infection (25.9%), intra-abdominal abscess (14.8%), infective endocarditis (11.1%), septic pulmonary emboli (11.1%), and gastrointestinal infection (11.1%) [2]. Predisposing factors for extraoral manifestations of *P. micra* include advanced age, immunocompromised state, odontogenic infection, and gastrointestinal malignancy [2].

*F. necrophorum* is a Gram-negative anaerobic rod that colonizes the oropharynx and GI tract. It is most widely known as a causative pathogen in Lemierre's syndrome [1]. In Lemierre's syndrome, preexisting oropharyngeal or odontogenic infection extends to the lateral pharyngeal space to invade the carotid sheath, subsequently causing suppurative jugular thrombophlebitis [1]. *F. necrophorum* is also known to cause peritonsillar abscesses, periodontal disease, septic arthritis, and rarely CNS infection and endocarditis [1]. *Fusobacterium* bacteremia is rare, comprising less than 1% of all bacteremia and less than 10% of anaerobic bacteremia [1]. There are case reports of hepatic abscesses caused by *Fusobacterium*, most of which are caused by hematogenous spread from either odontogenic infection or inflammation of the GI tract [3].

Liver abscesses in general are caused by a variety of pathogens [4]. The most common predisposing factor for liver abscess formation is underlying malignancy (52% of cases), most commonly hepatobiliary or pancreatic malignancy [5]. In a retrospective study performed in 2012 of pyogenic liver abscesses, *Klebsiella pneumonia* was found to be the most common pathogen aspirated from liver abscess (35.7%), followed by *E. coli* (25%), and *Pseudomonas aeruginosa* (17.8%) [4].

## 2. Case

A 36-year-old male with no prior medical history presented with two days of fever, cough, and headaches. He had returned to the US from southern Germany two days prior. The patient was febrile to 101.1°F and tachycardic at 120 beats per minute (bpm), but physical exam was otherwise benign. His COVID-19 test was negative. He was discharged home.

One week later, the patient returned with persistence of his symptoms. He was again febrile to 102.1°F and tachycardic at 122°bpm and his repeat COVID-19 test was negative. He had a leukocytosis of 18.1 k/*μ*L with 9.9% lymphocytes with neutrophil predominance of 80.3%. Lab work in the ER was also notable for elevation in his liver-associated enzymes, aspartate aminotransferase (AST) of 48 U/L, alanine aminotransferase (ALT) of 90 U/L, alkaline phosphatase of 196 U/L, and total bilirubin of 1.11 mg/dL. On review of records, the patient had historically normal liver function test. The patient denied any abdominal pain at this time. His blood cultures demonstrated no growth. The patient was offered admission for further imaging and workup of fever of unknown origin; however, he declined and opted for close outpatient follow-up.

Nine days later, the patient returned due to persistence of fevers, in addition to new nausea and diarrhea. Repeat COVID-19 test was negative. He was again tachycardic at 117 bpm but afebrile and was sent home with return precautions.

**Figure 1:**
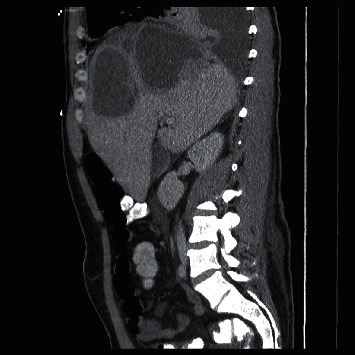
CT chest/abdomen/pelvis with contrast, sagittal view, obtained on hospital day 1 showing large hepatic abscesses.

Two weeks later, he presented with a fever of 103.3°F and was tachycardic at 138 bpm and hypotensive at 91/53 mmHg. He additionally endorsed fatigue, diarrhea, and shortness of breath. On physical exam, he was pale and ill appearing. He had palpable hepatosplenomegaly. His labs showed a leukocytosis of 22.1 k/*μ*L with 88.4% neutrophils. His AST was 64 U/L, ALT was 97 U/L, alkaline phosphatase was 411 U/L, and total bilirubin was 1.28 mg/dL. An abdominal CT scan was performed showing multiple large rim enhancing hepatic abscesses (Figure 1) ranging 8–11 cm in diameter, in addition to a right pleural effusion. The patient was admitted to the ICU for septic shock initially requiring vasopressor support. Initial empiric antibiotic regimen was vancomycin, piperacillin-tazobactam, and doxycycline.

After hemodynamic stabilization, thoracentesis was performed and interventional radiology (IR) placed hepatic drains. Three pigtail drains were placed in the largest hepatic cysts and two pigtail drains were placed for his right pleural effusion. Once stabilized, the patient was able to provide supplemental history that he denied recent travel to Central and South America, contact with sheep or sheep dogs, camping, and drinking unfiltered water. Given the broad infectious differential, a myriad of labs were ordered to help determine etiology and cause of his prolonged fever and underlying hepatic abscesses. His infectious workup included respiratory and GI viral polymerase chain reaction (PCR) panels, urine streptococcus and legionella antigens, malaria smear and rapid antigen, Lyme titers, stool culture, hepatitis panel, Epstein–Barr virus PCR, Cytomegalovirus PCR, Acid Fast Bacilli culture/smear, syphilis testing, human immunodeficiency virus antibody (Ab), Gonococcal, Chlamydia, Coxiella titers, Entamoeba antigen, stool ova and parasites, and repeat SARS-CoV-2 antibody testing which were all negative.

Blood cultures were obtained on hospital day 1. Cultures of hepatic cystic fluid were drawn on hospital day 2, once hepatic drains were placed. By hospital day 5, there was no growth on his wound or blood cultures. To help make a microbiologic diagnosis, his remaining sample from his hepatic wound culture was sent for broad range bacterial polymerase chain reaction. His blood cultures were asked to be held for 10 days in case his blood cultures had a fastidious organism. On hospital day 6, his blood cultures become positive. On hospital day 9, his cultures showed growth on his anaerobic plates only. Identification of his blood and abscess cultures was made using matrix-assisted laser desorption ionization time-of-flight mass spectrometry (MALDI-TOF). His blood culture grew *P. micra*. His wound cultures grew both *P. micra* and *F. necrophorum*. His broad range PCR later confirmed that the only microbes present were *P. micra* and *F. necrophorum*. Unfortunately, the hospital lab did not have the capability to perform antibiotic susceptibility testing on anaerobic bacteria. However, his *P. micra* abscess culture had a positive cefinase disk (chromogenic beta-lactamase test). Upon culture speciation, antibiotic regimen was transitioned to a 6-week course of ertapenem because of its high bioavailability and coverage for bacteremia from hepatic abscesses by anaerobic bacteria with beta-lactamase activity.

In an effort to identify the source of his infection, echocardiogram, colonoscopy, and esophagogastroduodenoscopy (EGD) were performed during admission. Echocardiogram showed no evidence of endocarditis. EGD and colonoscopy did not identify any malignancy or GI tract abnormalities. He also underwent dental evaluation while hospitalized, including dental X-rays that showed no evidence of odontogenic infection. The patient was discharged from the hospital after 17 days and completed his 6-week course of IV ertapenem at home. During a postdischarge dental exam, he was found to have a single dental cavity and he admitted to having dental caries that were repaired prior to returning from Germany.

**Figure 2:**
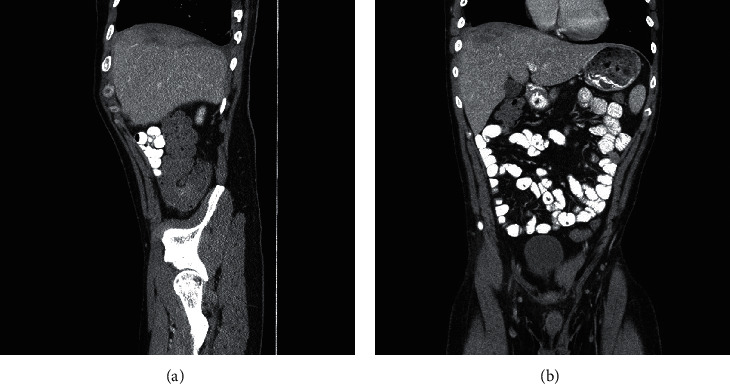
CT chest/abdomen/pelvis with contrast, sagittal view (a) and coronal view (b), 57 days after hospital admission, showing resolution of hepatic abscesses.

His repeat CT scan 2 months after discharge showed resolution of the abscesses with residual scar tissue in the superior aspect of the right lobe of the liver (Figures 2(a) and 2(b)). At this time, his ALT remained slightly elevated at 47 U/L, but AST, total bilirubin, and alkaline phosphatase had normalized. He completed a capsule endoscopy with no small bowel findings that could be the source of his infection.

## 3. Discussion

There are three reported cases of *P. micra* causing hepatic abscesses and none with a mixture *Parvimonas* and *Fusobacterium* [3, 6–14]. Our case was similar in presentation with other studies, with chief presenting symptoms of fever, chills, GI upset, and abdominal pain, worsening over a course of several weeks prior to diagnosis [6, 7, 14]. Previous case studies of *P. micra* abscesses describe a diverse spectrum of patients affected, to include a 90-year-old female in the setting of malignancy, a 54-year-old male with acute diverticulitis, and a 65-year-old female with concomitant brain abscesses [6, 7, 14].

There have been reported cases of young, otherwise healthy patients getting hepatic abscesses from *Fusobacterium*. The most common initial symptoms described in these cases are fever, chills, and diarrhea, ranging from 3 to 14 days prior to presentation [8, 13, 15]. Case reports include an otherwise healthy 21-year-old male, a 54-year-old male with concomitant lung abscesses and acute diverticulitis, and a 76-year-old female with preceding *Fusobacterium* bacteremia [8, 13, 15]. *Fusobacterium* is associated with periodontal disease, and one case report suggests that even routine dental cleaning can lead to *Fusobacterium* bacteremia and abscess formation [9]. Fortunately in these specific case reports, each patient clinically improved after abscess drainage and antibiotic therapy [8, 9, 13, 15].

Based on literature review, coinfection of Parvimonas and Fusobacterium appears to be extremely rare. One reported case involved a 39-year-old male with psoriatic arthritis who had recently initiated immunosuppressive therapy and presented with new-onset seizures [16]. He was subsequently found to have a brain abscess, which when cultured grew *P. micra*, *F. nucleatum*, and *S. sanguinis* [16]. It was later discovered he had undiagnosed periodontal disease prior to starting immunosuppressive therapy, thought to be the origin of his infection [16]. The second reported case involved a 72-year-old female with metastatic breast cancer, vertebral metastasis, and destructive spondylodiscitis with intraspinal abscess [17]. Her abscess culture grew *P. micra* and *F. nucleatum* [17]. In both cases, abscess drainage and antibiotic therapy successfully resolved the infection [16, 17].

The treatment of hepatic abscesses was similar throughout the case reports, all requiring IV antibiotics and some involving percutaneous drainage. Duration of treatment was anywhere from a few weeks to 6 months [11]. Most cases required 6–8 weeks of treatment with various antibiotic, including beta-lactams, carbapenems, metronidazole, and sometimes fluoroquinolones [11]. Of the cases reviewed, most of the patients cleared the infections 3–12 weeks after antibiotic initiation, based on CT scans. One patient was put on comfort care with only 2 days of IV antibiotics and died 12 days later while a second reported death did not discuss the circumstances [3, 6–15]. Our case was similar in both treatment and prognosis with percutaneous drainage and 6 weeks of ertapenem, leading to complete resolution at 2 months. Additionally, ertapenem was chosen for because of its high bioavailability and coverage for bacteremia from hepatic abscesses by anaerobic bacteria with beta-lactamase activity. Ertapenem was chosen over metronidazole in order to avoid the potential complication of neuropathy that can occur with longer courses [18].

Both *Parvimonas* and *Fusobacterium* have been reported to cause hepatic abscesses individually. However, to the authors' knowledge, this is the first documented hepatic abscess coinfection of these two organisms [3, 6, 7, 19]. This is also one of the few cases reported of hepatic abscess involving these bacteria in an otherwise healthy young male. Though these bacteria are known to colonize the alimentary tract, it is unlikely for them to cause multiple liver abscesses spontaneously in this demographic [2, 15]. It is difficult to tell the etiology of our patient's infection, though his history of dental caries remains the most likely source. Additionally, it is worth noting that anaerobic bacteria are difficult to culture, and multiple anaerobes causing a hepatic abscess may be more common in clinical practice than what is currently reported in the literature. This case would still represent one of very few documented cases of hepatic abscesses from either organism in an immunocompetent healthy young adult.

## Data Availability

No data were used to support this study.
